# Influence of edge enhancement applied in endoscopic systems on sharpness and noise

**DOI:** 10.1117/1.JBO.27.10.106001

**Published:** 2022-10-06

**Authors:** Geert Geleijnse, Bernd Rieger

**Affiliations:** a Erasmus University Medical Center, Department of Ear, Nose, & Throat, Rotterdam, The Netherlands; b Delft University of Technology, Department of Imaging Physics, Faculty of Applied Sciences, Delft, The Netherlands

**Keywords:** edge enhancement, sharpening, endoscopy, modulation transfer function, noise

## Abstract

**Significance:**

Flexible endoscopes are essential for medical internal examinations. Digital endoscopes are connected to a video processor that can apply various operations to enhance the image. One of those operations is edge enhancement, which has a major impact on the perceived image quality by medical professionals. However, the specific methods and parameters of this operation are undisclosed and the arbitrary units to express the level of edge enhancement differ per video processor.

**Aim:**

Objectively quantify the level of edge enhancement from the recorded images alone, and measure the effect on sharpness and noise.

**Approach:**

Edge enhancement was studied in four types of flexible digital ear nose and throat endoscopes. Measurements were performed using slanted edges and gray patches. The level of edge enhancement was determined by subtracting the step response of an image without edge enhancement from images with selected settings of edge enhancement and measuring the resulting peak-to-peak differences. These values were then normalized by the step size. Sharpness was characterized by observing the normalized modulation transfer function (MTF) and computing the spatial frequency at 50% MTF. The noise was measured on the gray patches and computed as a weighted sum of variances from the luminance and two chrominance channels of the pixel values.

**Results:**

The measured levels were consistent with the level set via the user interface on the video processor and varied typically from 0 to 1.3. Both sharpness and noise increase with larger levels of edge enhancement with factors of 3 and 4 respectively.

**Conclusions:**

The presented method overcomes the issue of vendors expressing the level of edge enhancement each differently in arbitrary units. This allows us to compare the effects, and we can start exploring the relationship with the subjectively perceived image quality by medical professionals to find substantiated optimal settings.

## 1 Introduction

**Fig. 1:**
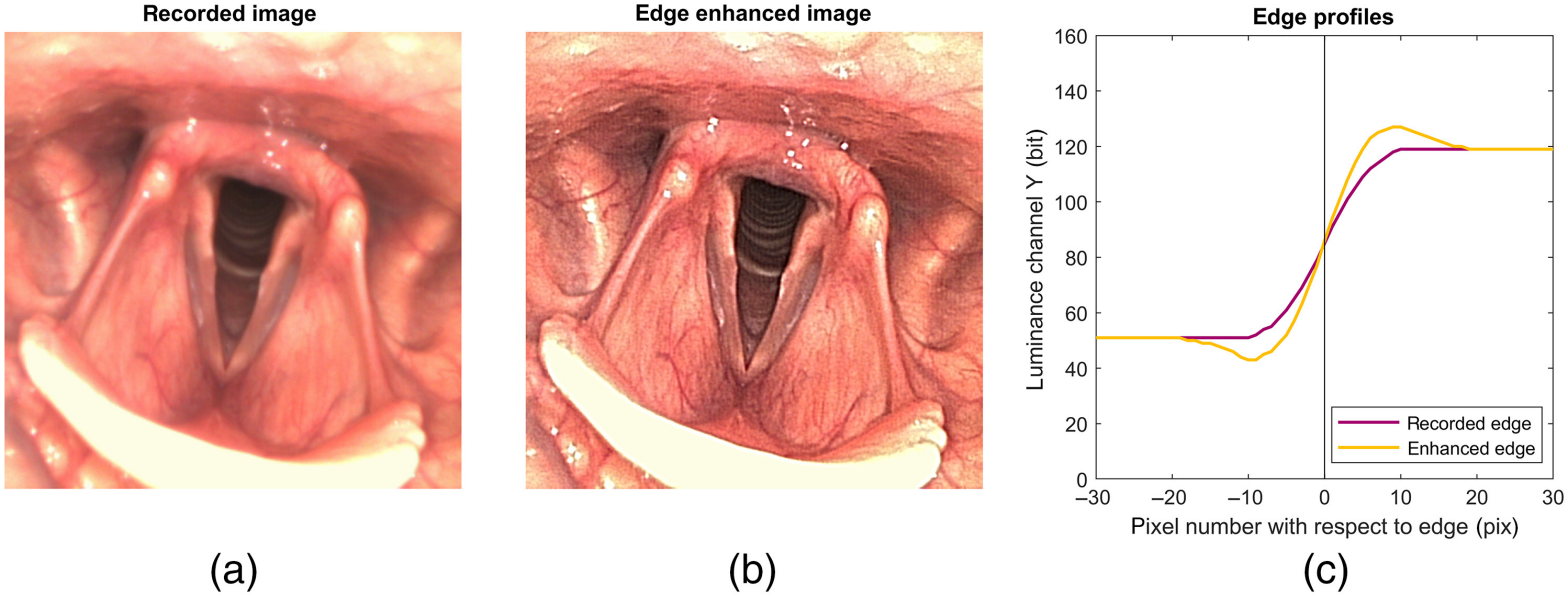
(a) Example image of a larynx without edge enhancement. (b) Edge enhancement applied to the example image of the larynx. (c) Profiles of a stimulus edge to an endoscope. The recorded edge is spread over different pixels due to the bandlimit and improper sampling of an endoscopic system. Edge enhancement is applied to the recorded edge to illustrate the added undershoot on the darker side of an edge and an overshoot on the brighter side.

Flexible endoscopes are essential to examine nose, throat, and upper airway.^1^ In former days, these were fiber-optic endoscopes offering an image that was observed directly with the eyepiece or by a small camera that was connected to it. Fiber-optic endoscopes have gradually been replaced by digital endoscopes because of much better image quality.^2^^–^^6^ Digital endoscopes are connected to a video processor that can apply various operations to enhance the image without perceivable delay for the observer. One of those operations is edge enhancement and its effect on an *in vivo* image of the larynx is shown in Fig. 1. This operation makes the image sharper and sharpness is strongly correlated with the perceived image quality by ear nose and throat (ENT) professionals.^7^ Although edge enhancement is applied by all vendors, the literature on edge enhancement in ENT is limited. Kawaida et al.^8^ reported that in their experience image quality was improved when structure enhancement, i.e., a form of edge enhancement, was applied. Kawaida et al.^9^ later showed that edge enhancement also seems to improve diagnostic accuracy: applying structure enhancement changed the diagnosis in 2 out of 15 patients.

Edge enhancement or sharpening is a known technique to sharpen edges by adding an undershoot on the darker side of an edge and an overshoot on the brighter side.^10^^–^^12^ In fact, this operation does not introduce new information to the image but increases the step in brightness of edges. This operation probably works so well, because it mimics the biological process of retinal lateral inhibition in the visual system.^13^ A major drawback of edge enhancement is that the operation cannot discern edges from noise and therefore enhances both.

Common methods to apply edge enhancement are unsharp masking and Laplacian of a Gaussian.^11^^,^^14^ These methods both use two parameters: radius and amount that are optimized for the system and its application. The radius indicates the area that is involved for the enhancement and the amount is the strength of the enhancement. To prevent noise to be increased, edge enhancement algorithms can have a minimum contrast level setting that prevents low contrast noise to be enhanced. Consequently, low-level edges will not be enhanced as well. Numerous variations to these typical methods have been developed for esthetic, functional, and diagnostic purposes.^10^^,^^11^

Olympus, Pentax, Xion, and Storz offer ENT-endoscopes that apply edge enhancement, but the specific method and parameters are not disclosed.^15^ Literature to substantiate the default settings has not been found either. Since we do not know the methods and parameters applied by the vendors, we can only measure the effects on images that are processed by the video processors. Pentax and Olympus allow the user to adjust the level of edge enhancement, although the units to express the level of edge enhancement are arbitrary and differ, e.g., Olympus CV-170 uses nine levels A0–A8, and Pentax VIVIDEO CP-1000 has a bar without a numeric indicator.

The purpose of this study is to: (1) objectively quantify the level of edge enhancement from the images alone, and (2) measure the effect of edge enhancement on sharpness and noise.

## 2 Methods

We included four ENT-endoscopic systems with the option to adjust the level of edge enhancement that was applied by the video processor. Three of those video processors can also be applied for urological and gastroenterological purposes if appropriate types of endoscopes are connected to the video processor. The level of edge enhancement was increased from zero to maximum in discrete steps. The number of steps depended on the available settings of the system under test (Table 1).

**Table 1:** Included endoscopy systems. Endoscope type as specified by the manufacturer. Endoscope diameter measured with a Mitutoyo caliper. Video processor type as specified by the manufacturer. Number of pixels effectively used to display the view of the endoscope. Manufacturer. The term used by the manufacturer for edge enhancement.

Endoscope	Diameter (mm)	Video processor	Pixels (horz × vert)	Manufacturer	Term used for edge enhancement
ENF-V4	2.9	CV-170	1080×1080	Olympus	Structure enhancement
ENF-VH	4.4	CV-170	1730×1080	Olympus	Structure enhancement
VNL9-CP	3.2	VIVIDEO CP1000	800×800	Pentax	Edge enhancement
VNL11-J10	4.5	DEFINA EPK-3000	1175×900	Pentax	Visual enhancement

### 2.1 Image Acquisition

**Fig. 2:**
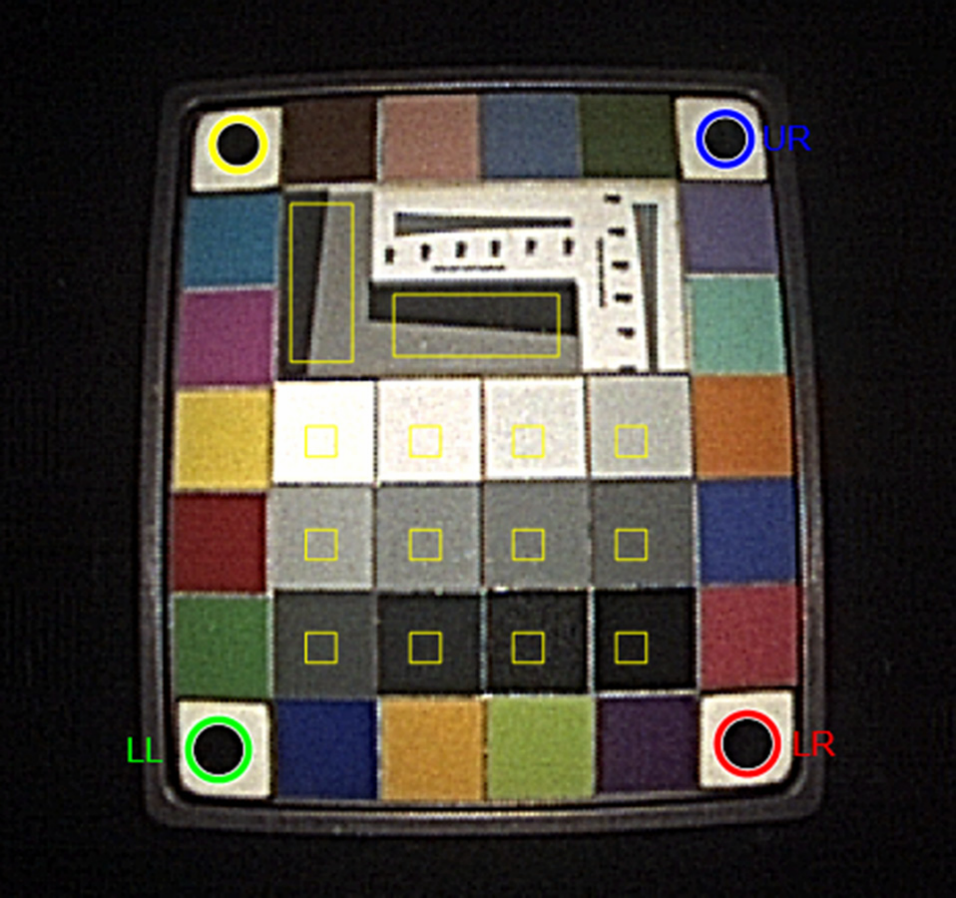
Image of the test target Rez checker target matte acquired with a Pentax VNL9-CP. This chart is 2.54×2.22  cm in size. The analysis software located the black circles in the corners of the target. The red, green, and blue circles are positioned lower left (LR), lower left (LL) and upper right (UR), respectively. The coordinates of these circles were used as reference to determine the regions of interest. The yellow circles are detected circles that are not being used. The yellow squares indicate the regions of interest for analysis. For this manuscript, we only used the gray patches and the slanted edges. The horizontal and vertical step responses were measured at the vertical and horizontal slanted edge, respectively.

The test target was a Rez checker target matte (Imatest^®^, Boulder, Colorado), a test chart that was designed by Image Science associates to be more suited to narrow illumination geometries such as those used in endoscopic imaging (see Fig. 2).^16^ The manufacturers of the ENT-endoscopes specify an operating range of 5 to 50 mm, but the focal length is undisclosed. The tip of the endoscope was positioned at a distance of 30 mm to the target, because ENT-professionals estimated this as the common operational distance. In case an abnormality is seen, the ENT-professional will try to maneuver the tip closer for a better view, but this is not always tolerated by the patient, resulting in gag reflexes or other patient discomfort.

During testing, the endoscope tip was then rotated such that the edges on the test image were oriented horizontally or vertically. The flexible tip was bent using the endoscope handle to center the target mid screen. The light source of the endoscopic system was used to illuminate the target and white balance was performed using an RAL9003 test chart temporarily positioned in front of the tip. Care was taken that no ambient light fell on the test chart during white balancing. The illumination of the target was adjusted so that the brightest gray patch on the target showed signs of pixels being saturated. This way the other gray patches were available for measuring the optoelectronic conversion function (OECF) and the measured step response on the soft contrast slanted edge is within the dynamic range of the system. When the level of edge enhancement is increased, the step response shows an undershoot and overshoot that should not be clipped.^17^ The step response was used to determine the level of edge enhancement and modulation transfer function (MTF) as described in the following paragraphs.

The images were captured using an Epiphan DVI2USB3 frame grabber connected to the DVID-D output of the endoscope video processor and stored as 24-bits per RGB pixel bitmap files to prevent compression.

### 2.2 Image Analysis

A custom-written MATLAB program (R2022a, The MathWorks Inc., Natick, Massachusetts) identified the circles on the target image automatically (see Fig. 2), such that the regions of interest can be further processed (yellow rectangles in Fig. 2). The image was mainly analyzed according to ISO12233:2017^16^ Additional unidirectional low-pass filtering was introduced for the edge detection to avoid reflections from perturbing the localization of the slanted edge.

### 2.3 Step Response of the System

The horizontal and vertical step responses of the system and the OECF that is required for linearization were measured on the slanted edges and the gray patches in the middle as indicated by the yellow boxes in Fig. 2. The RGB-values within the region of interest (ROI) were converted to luminance values Y using the following formula:

Y(R,G,B)=0.2125·R+0.7154·G+0.0721·B, (1)

where R,G, and B are the red, green, and blue values, respectively.

**Fig. 3:**
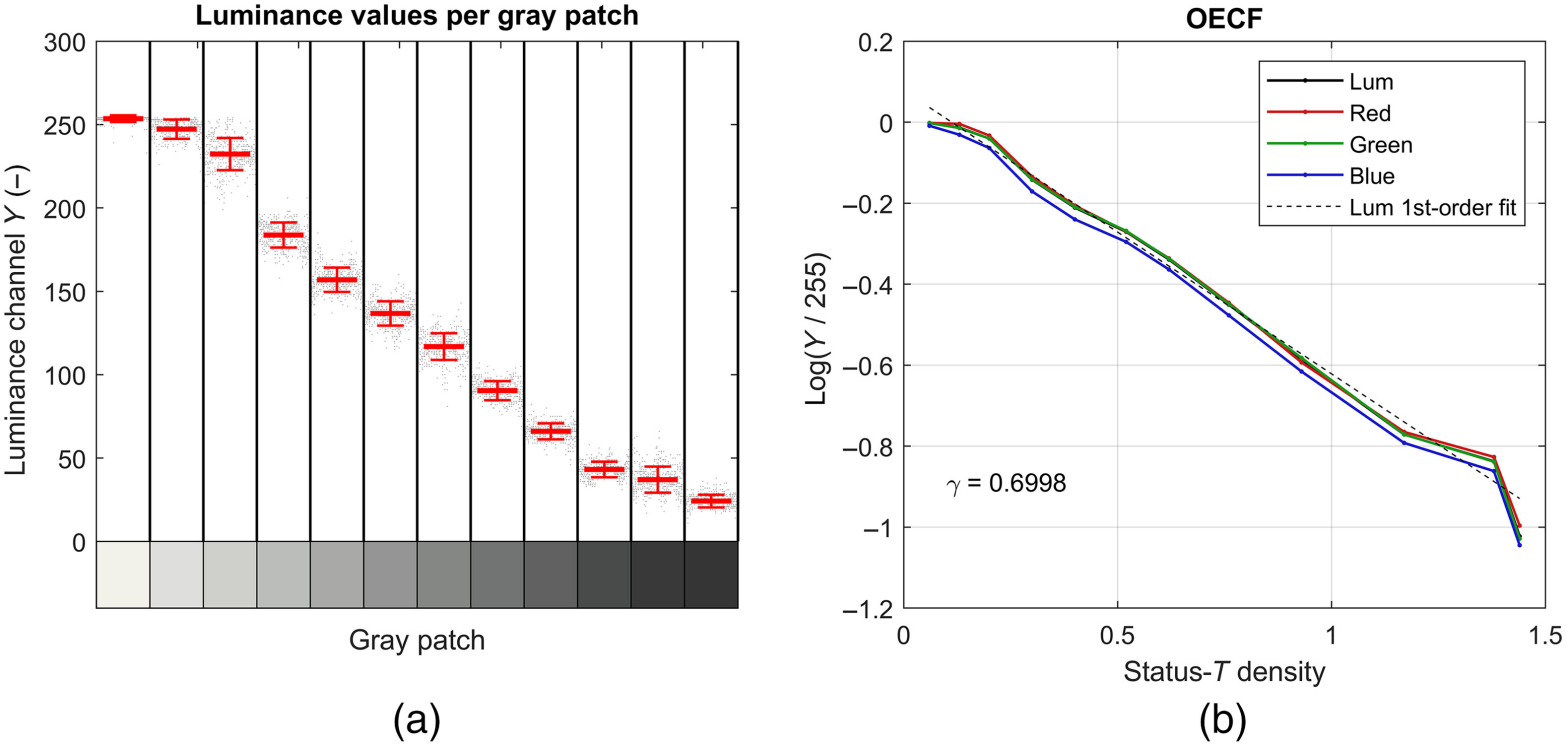
(a) Measured luminance values per gray patch. The luminance values of the pixels in the regions of interest on the gray patches are plotted individually with small gray dots. The mean luminance values are plotted with a wide horizontal line per gray patch. The smaller red horizontal lines indicate the standard deviations. (b) OECF. Luminance values plotted versus the status-T density for estimating the slope of the linear fit. Red, green and blue coincide, indicating a good white balance.

The gamma value of the endoscopic system was estimated from the pixels within the ROI on the gray patches in the middle (Fig. 2). The RGB-values were converted to luminance values and averaged per ROI. The size of the ROI was tuned to avoid border effects and luminance variation within one ROI. An example of the measured luminance values and mean values per gray-patch is shown in Fig. 3(a). The averages of the measured luminance values and status-T densities of the patches as specified by image science associates were used to measure the gamma-value of the endoscope system [Fig. 3(b)],

$$\log_{10}(Y/255)=-\gamma \cdot T+C,$$ (2)

in which Y is the luminance value, 255 is the maximum value of an 8-bit number per color (RGB 3×8=24 bits per pixel), γ is the gamma value, T the status T-density, and C a constant of the linear fit. Status-T density is the wide band color reflection density: $T=\log_{10}(1/R)$, e.g., T=1 indicates a reflectance of 10%. To prevent saturated patches underestimating the gamma value, we only used the patches with a luminance value above the minimum value plus 10% range and below maximum minus 10% range for the linear fit. The range was defined as the difference between the minimum and maximum luminance value.

The luminance response in the ROI with the horizontal slanted edge was linearized using:

$$Y_{\mathrm{lin}}(Y)=255\cdot (Y/255{)}^{\frac{1}{\gamma }}.$$ (3)

**Fig. 4:**
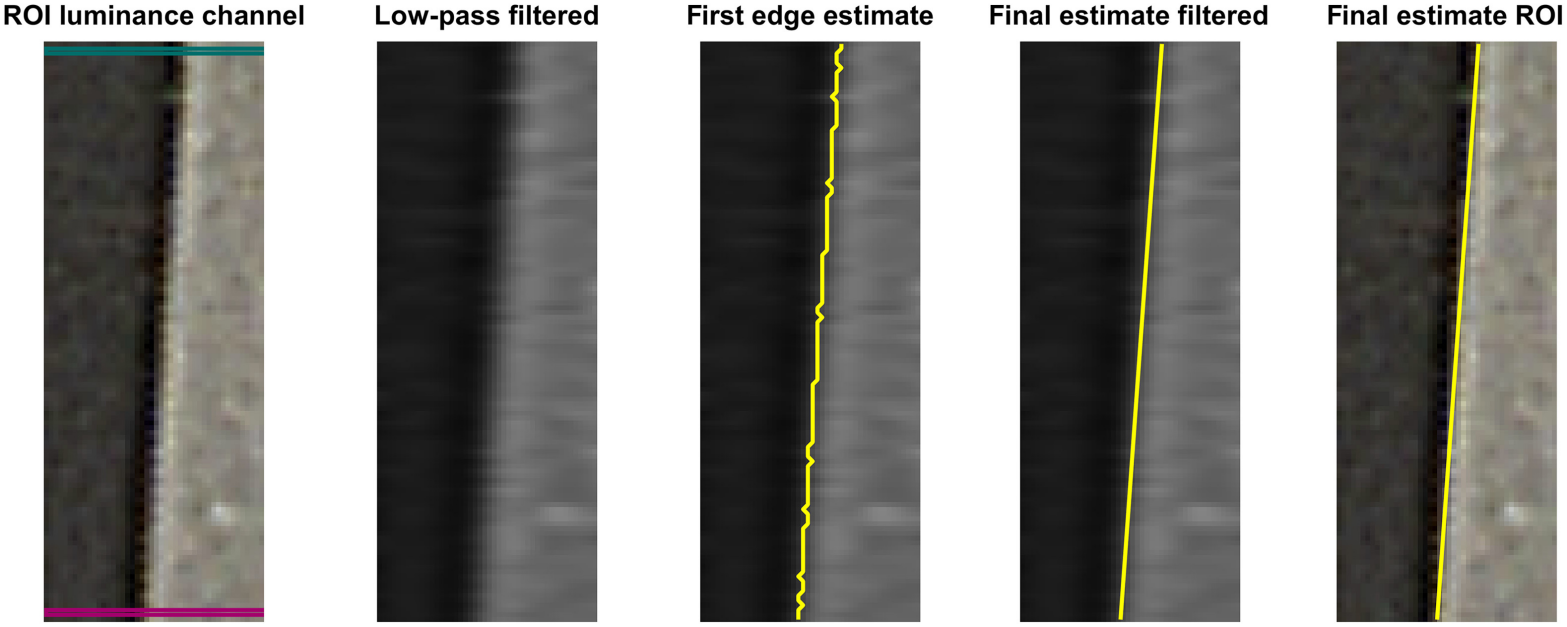
The slanted edge is located using the following steps. First, the ROI RGB-values are converted to luminance values and linearized. Second, each pixel line is low-pass filtered to avoid reflections from disturbing the first estimate of the slanted edge location. Third, the maximum gradient of each pixel line was used as a first estimate of the edge location. The final estimate is obtained with a linear fit to the first estimate. Last, the obtained edge location is plotted over the unfiltered ROI for visual inspection. The luminance values on the pink and green lines are plotted in Fig. 5(a).

Having the ROI with the slanted edge converted to luminance values and linearized (Fig. 4), the edge was detected. First, each data line of pixels in the ROI crossing the slanted edge was low-pass filtered using a unidirectional filter to avoid reflections within the ROI to disturb the edge detection. The maximum gradient on each data line of the filtered image was used as the first estimate of the edge. The final estimate was obtained by a linear fit on the first estimate. The final estimate of the edge was then plotted on the unfiltered image for a visual check.

**Fig. 5:**
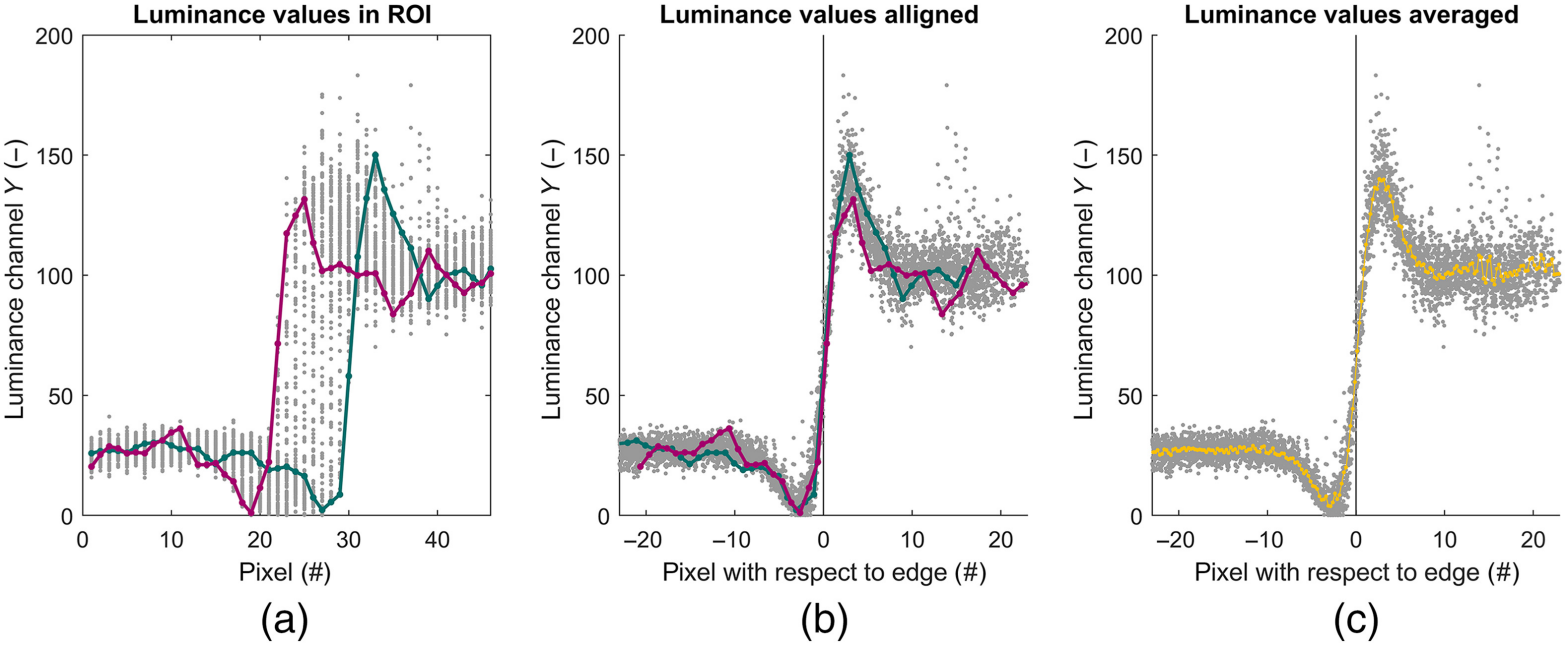
(a) The pixel luminance values $Y_{\mathrm{lin}}$ plotted versus their pixel distance to the slanted edge. The pink and green traces refer to the pink and green line in Fig. 4 left. (b) The step responses are aligned by the estimated edge location. The luminance values are plotted versus the perpendicular distance to the slanted edge. (c) The luminance values are averaged within each bin of ¼ of a pixel.

The step responses for each pixel line [Fig. 5(a)] were aligned using the distances of the pixels within the ROI perpendicular to the estimated slanted edge [Fig. 5(b)]. The luminance values were then binned into four bins per pixel (4× upsampling) relative to the edge and averaged per bin, yielding the step response of the system [Fig. 5(c)].

### Level of Edge Enhancement

**Fig. 6:**
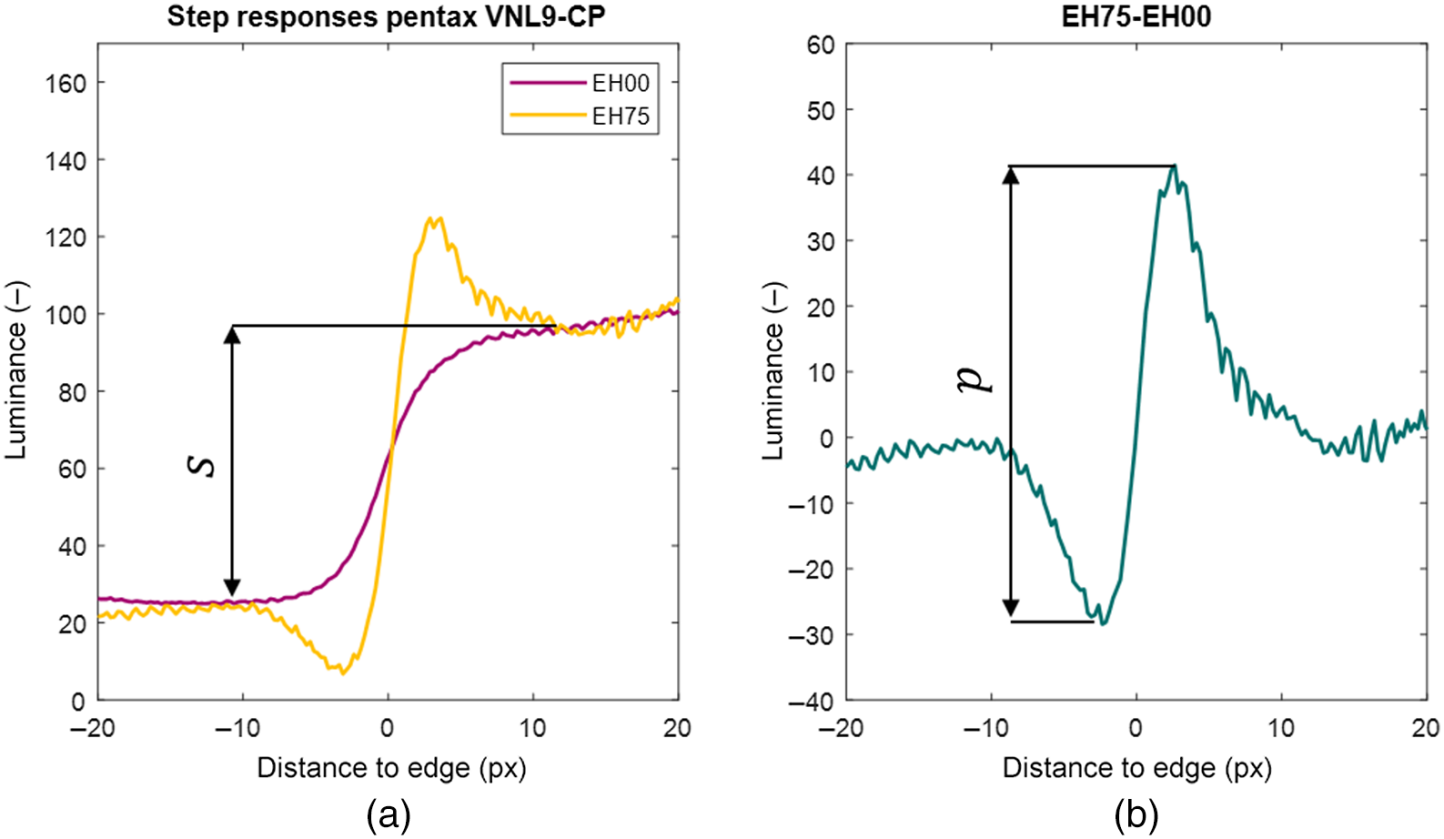
(a) Step responses of the Pentax VNL9-CP without edge enhancement (EH00) and with 75% edge enhancement (EH75) as indicated on the Pentax ViVideo CP-1000 video processor. The arrow indicates the step size s. (b) Difference between 75% edge enhancement step response minus 0%. The arrow indicates the peak-to-peak amplitude p.

Figure 6(a) shows two-step responses: one without edge enhancement (EH00) and another with some degree of edge enhancement (EH75). Since the algorithms and their parameters to perform the edge enhancement operation are undisclosed, we had to measure the level of edge enhancement. First, we subtracted the unenhanced from the enhanced step response and measured the distance between the minimum and maximum value p [Fig. 6(b)]. The peak-to-peak amplitude is dependent on the step size, therefore, the step size of the edge  s is measured as the difference between luminance values where the enhanced and unenhanced step responses converge Fig. 6(a). Then the level of edge enhancement is quantified by the peak-to-peak p amplitude divided by the step size s as L=(p/s).

### 2.5 Sharpness (MTF50 and MTF50P)

**Fig. 7:**
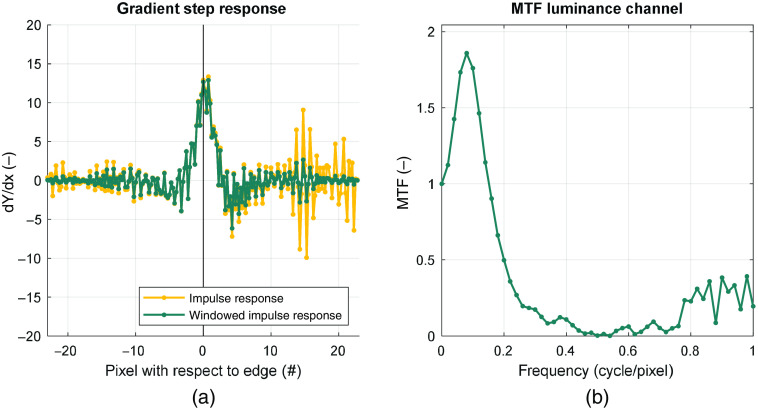
(a) The impulse response (yellow) is obtained by differentiating the step response. A Hamming window is applied to get the windowed impulse response (green). The window function suppresses noise far from the edge location. (b) The impulse response is Fourier transformed and normalized to the contrast at zero frequency to get the MTF.

Sharpness is the ability of a system to transfer contrast at certain spatial frequencies and is expressed by the MTF. To obtain the MTF, the step response is numerically differentiated to get the impulse response of the system [Fig. 7(a)]. A Hamming window was applied to reduce noise from reflections located far from the slanted edge and make the waveform periodic to prevent spectral leakage. Finally, the MTF was obtained by Fourier transformation of the impulse response, calculating the modulus, and normalizing by the contrast at zero frequency [Fig. 7(b)].

**Fig. 8:**
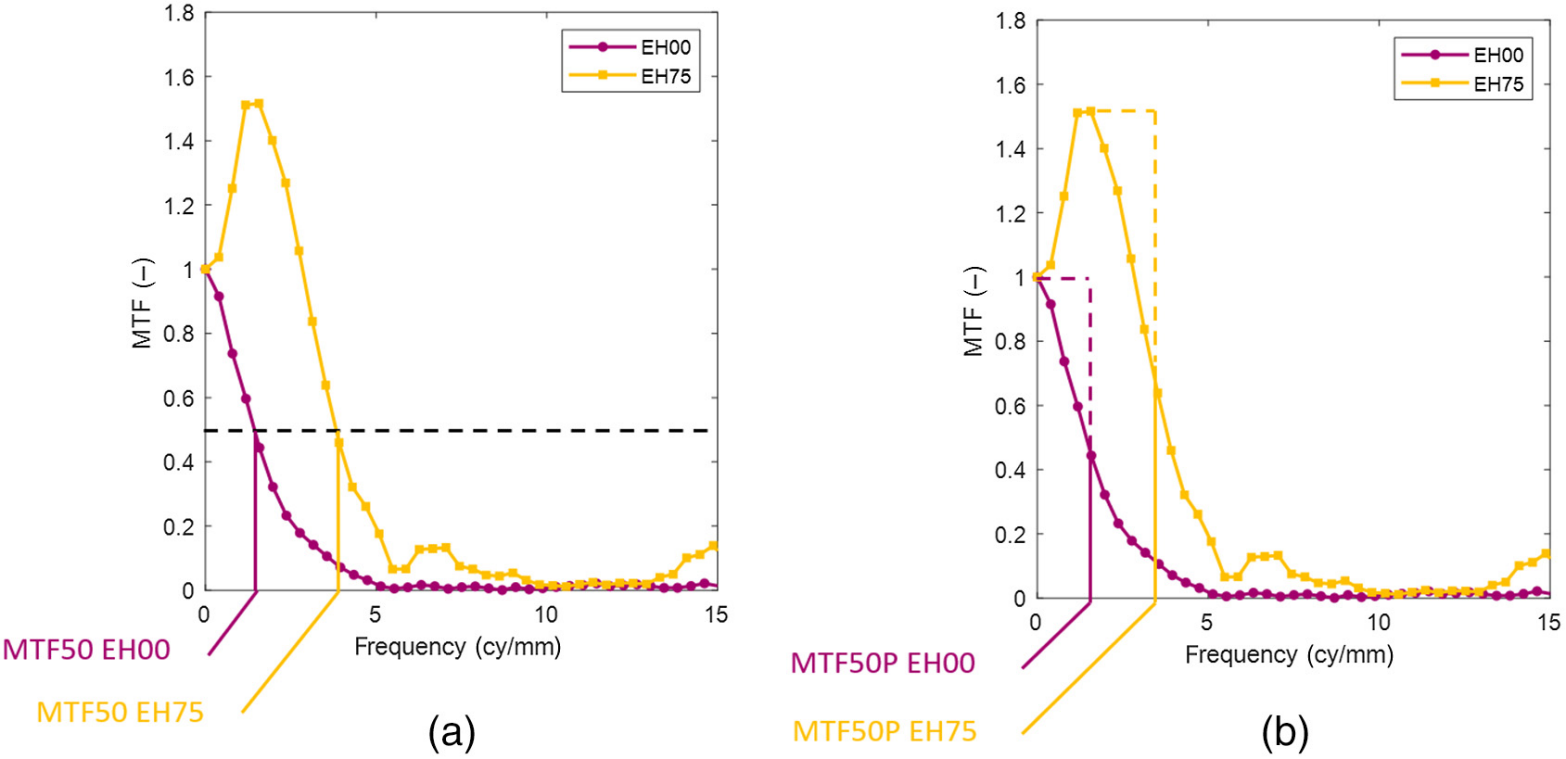
(a) MTF curve of the Pentax VNL9-CP without edge enhancement and 75% of maximum edge enhancement. MTF50 is the frequency at which the MTF curve first crosses the 0.5. (b) MTF50P is the frequency at which the MTF curve first crosses the 0.5 times the peak value.

The MTF frequency is expressed in cycles per pixel [horizontal axis Fig. 7(b)]. To calculate the frequency in cycles per unit distance (Fig. 8), the number of pixels between the centers of the black circles in Fig. 2 is measured horizontally and vertically. The number of pixels correspond to the physical horizontal distance of 16 mm and vertical distance of 19 mm.

When edge enhancement is applied by the video processor, the original unenhanced MTF curve is blown up. Above certain levels of edge enhancement, the peak will grow above 1 as can be seen in (Fig. 8). Ideally, we would measure and compare the complete MTF curve as the whole MTF characterizes how the spatial frequencies are transmitted by the system. Comparison of curves is more difficult than comparing a scalar, so we looked for a simple number. MTF50 is the spatial frequency at which the MTF crosses 0.5 and is determined by linear interpolation [Fig. 8(a)] and is commonly used as a summary metric for the MTF performance.^18^^,^^19^ Sometimes, other metrics are used such as MTF10. MTF10 is the frequency at which the MTF curve crosses 0.1. This approximates to the Rayleigh criterion for maximal resolution, at which frequency it is just possible to discern two lines.^17^^,^^18^ MTF10 would correlate to counting television lines. However, MTF10 is not robustly to measure since the signal is relatively close to the noise floor and the slope of the MTF curve is small. MTF50 is another point on the MTF curve that has a much better signal-to-noise ratio (SNR) and usually has a steep slope, making it more suitable for the purpose of this study. A disadvantage of MTF50 is that it is heavily influenced by edge enhancement. MTF50P is the spatial frequency at which the MTF crosses 0.5 of its peak value and is less sensitive to the effect of edge enhancement^18^ [Fig. 8(b)].

### 2.6 Noise

**Fig. 9:**
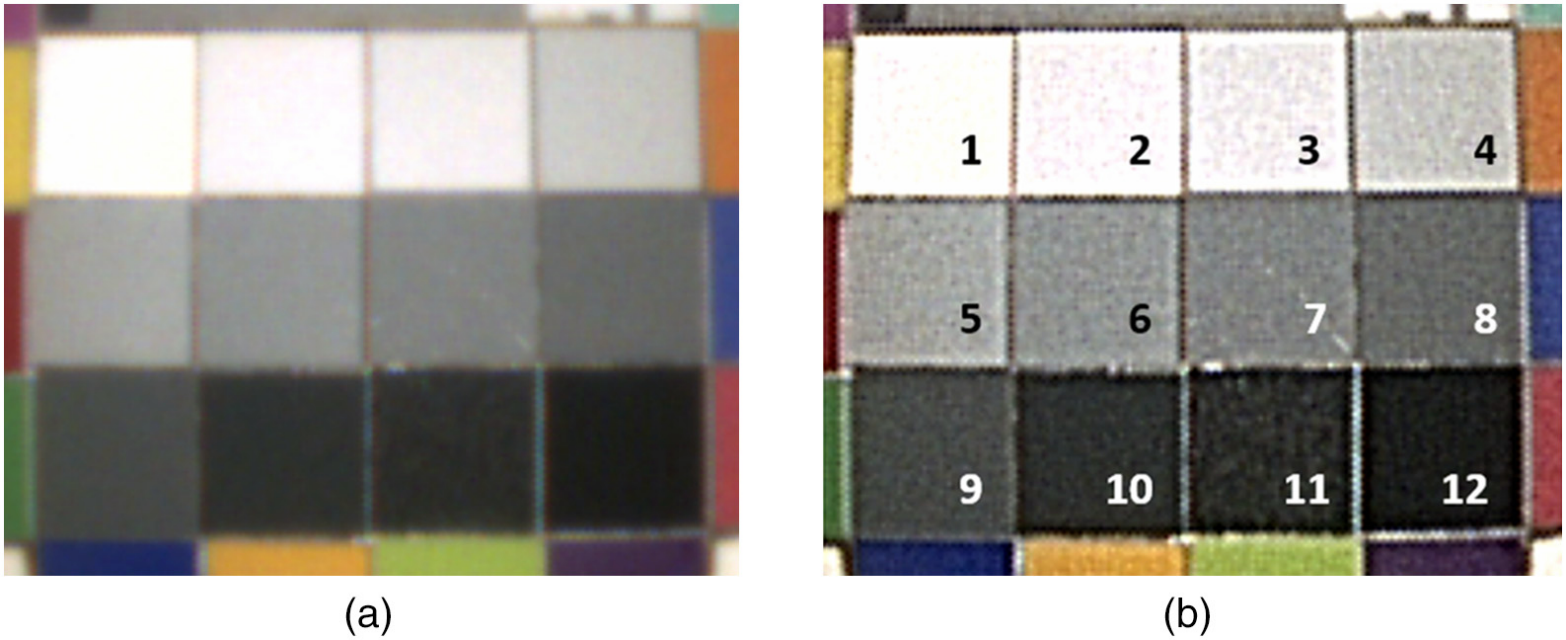
(a) VNL9-CP without edge enhancement and (b) with 75% edge enhancement as indicated on the VIVIDEO CP-1000 video processor. Image A is blurry and the gray patches are flat gray, whereas image B is sharper and the gray patches have more image noise. The numbering is added for referencing the gray patches in the results section.

Increasing the level of edge enhancement also increases the amount of noise [Figs. 9(a) versus 9(b)]. The noise could have been calculated as the root-mean-square of the luminance channel, however, the relative visual contributions to the perceived noise of the two chrominance signals would be missing. Therefore, we used the formula proposed by Kelly and Keelan,^20^^,^^21^

$$\sigma_{V}(Y,R,B)=\sqrt{\sigma (Y{)}^{2}+0.279\cdot \sigma (Y-R{)}^{2}+0.088\cdot \sigma (Y-B{)}^{2},}$$ (4)

in which σ(Y) is the standard deviation of the luminance values within the ROI as indicated in Fig. 2. σ(Y−R) and σ(Y−G) are the standard deviations of the luminance values minus the red and green values, respectively. The noise values were averaged over the gray patches. The gray patches with noise that showed saturation due to the limited dynamic range were excluded from the average.

The Rez checker target nano matte has gray patches with a fine texture to scatter light from the endoscope and prevent it from reflecting directly to the lens of the endoscope resulting in flare. This texture can interfere with noise measurements if the endoscopic system can resolve this texture and result in an overestimation of the noise. To determine if texture was observed, we positioned the tip of the endoscope at 20, 30, and 40 mm to the target and measured the noise at the individual gray patches using nine edge enhancement settings. If the measured noise on the specific gray patch varies with distance, the endoscopic system can resolve texture on that gray patch and it cannot be used for noise measurements.

### 2.7 Signal-to-Noise Ratio

The SNR was defined as

$$\mathrm{SNR}=20\cdot \log_{10}(\frac{Y_{\mathrm{snr}}}{\sigma_{\mathrm{snr}}}).$$ (5)

The SNR-values were calculated for all gray patches. Similar to ISO15739,^21^ the SNR-value was reported at one specific luminance level. First, the status-T density value ($T_{\mathrm{ref}}$) at which the luminance value was equal to gray value 240 was determined. This value was linearly interpolated between the adjacent data points on the measured OECF-curve [Fig. 3(b)]. Second, the status-T density value for the SNR-value was calculated

$$T_{\mathrm{snr}}=T_{\mathrm{ref}}-\log_{10}(0.13).$$ (6)

Last, the SNR-value at $T_{\mathrm{snr}}$ was determined by linear interpolation between the adjacent measured SNR-values.

## 3 Results

**Fig. 10:**
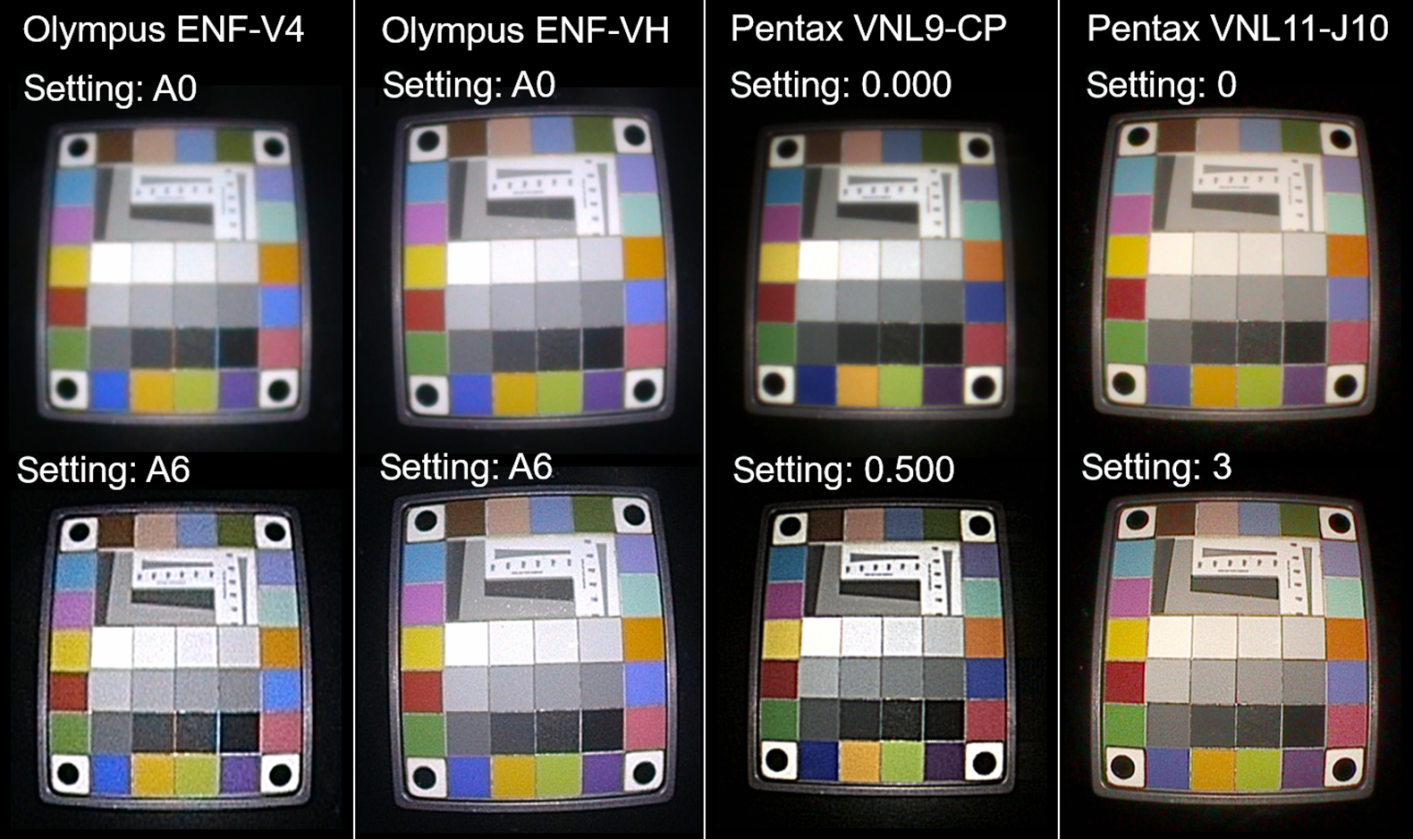
Example images of the Rez Checker Target Nano recorded with the four included endoscopes. Each quadrant contains two images per endoscope: the top image is recorded without edge enhancement and the bottom image is recorded with a level of edge enhancement closest to the 0.750 of the Pentax VNL9-CP. The black backgrounds of the images are cropped and the image size is scaled to show the test target.

Ten series of images have been acquired in this study (Table 2). The first eight series are acquired to demonstrate how the presented method can objectively quantify the level of edge enhancement of an endoscopic system and measure the effect of edge enhancement on sharpness and noise. The last two series have been added to illustrate the influence of the endoscope tip to target distance and show how texture on the gray patches can affect noise measurements. Example images recorded by the four included endoscopes with and without edge enhancement are shown in Fig. 10.

**Table 2:** List of analyzed test images. Endoscope type as specified by the manufacturer. Two independent test series were acquired by repositioning the endoscope in the setup. Distance between endoscope tip and test chart. Applied edge enhancement setting as specified in Table 1. Number of test images acquired.

Endoscope	Test/retest	Distance (mm)	Edge enhancement settings	Number of images
Pentax VNL9-CP	Test	30	0.000, 0.125, 0.250, …, 1.000	9
	Re-test	30	0.000, 0.125, 0.250, …, 1.000	9
Olympus ENF-V4	Test	30	A0, A1, A2, …, A8	9
	Re-test	30	A0, A1, A2, …, A8	9
Olympus ENF-VH	Test	30	A0, A1, A2, …, A8	9
	Re-test	30	A0, A1, A2, …, A8	9
Pentax VNL11-J10	Test	30	0,1, …, 6	7
	Re-test	30	0,1, …, 6	7
Pentax VNL-9 CP	Test	20	0.000, 0.125, 0.250, …, 1.000	9
Pentax VNL-9 CP	Test	40	0.000, 0.125, 0.250, …, 1.000	9
			**Total**	**86**

### 3.1 Level of Edge Enhancement

The step responses on the slanted edges have been analyzed to measure the level of edge enhancement L. In Fig. 10(a), the ratios of the horizontal level of edge enhancement $L_{x}$ and vertical level of edge enhancement $L_{y}$ are plotted versus the mean of $L_{x}$ and $L_{y}$. The line is a linear fit to the test and re-test data points. The increase of $L_{x}$ and $L_{y}$ was consistent with the edge enhancement settings in the user interfaces (Table 3). The Pentax VNL9-CP has fairly equally distributed levels of edge enhancement compared to the Pentax VNL11-J10, Olympus ENF-V4, and ENF-VH. The initial step of the Olympus ENF-V4 and ENF-VH from A0 to A1 is a relatively large step, but the other steps from A1 to A8 are equally distanced. Ratios of $L_{x}/L_{y}$ close to one indicate equal levels of horizontal and vertical edge enhancement. It can be seen that the ratio of the VNL9-CP is constantly slightly below one, meaning that $L_{y}$ is larger compared to $L_{x}$. The ratio of the VNL11-J10 is steadily increasing, meaning that $L_{x}$ grows faster compared to $L_{y}$ and the applied edge enhancement is anisotropic. The Olympus ENF-V4 and ENF-VH ratios are consistently positive and show considerable variation.

**Table 3:** Measured average levels of edge enhancement per edge enhancement setting listed for each type of endoscope.

Pentax VNL9-CP		Olympus ENF-V4		Olympus ENF-VH		Pentax VNL11-J10	
Setting	Level	Setting	Level	Setting	Level	Setting	Level
0.1250	0.16	A1	0.34	A1	0.28	1	0.27
0.2500	0.33	A2	0.42	A2	0.34	2	0.54
0.3750	0.48	A3	0.50	A3	0.42	3	0.85
0.5000	0.66	A4	0.57	A4	0.51	4	1.00
0.6250	0.77	A5	0.74	A5	0.59	5	1.30
0.7500	0.93	A6	0.86	A6	0.67	6	1.66
0.8750	1.07	A7	1.04	A7	0.84	—	—
1.000	1.27	A8	1.15	A8	1.11	—	—

### 3.2 Sharpness (MTF50 and MTF50P)

**Fig. 11:**
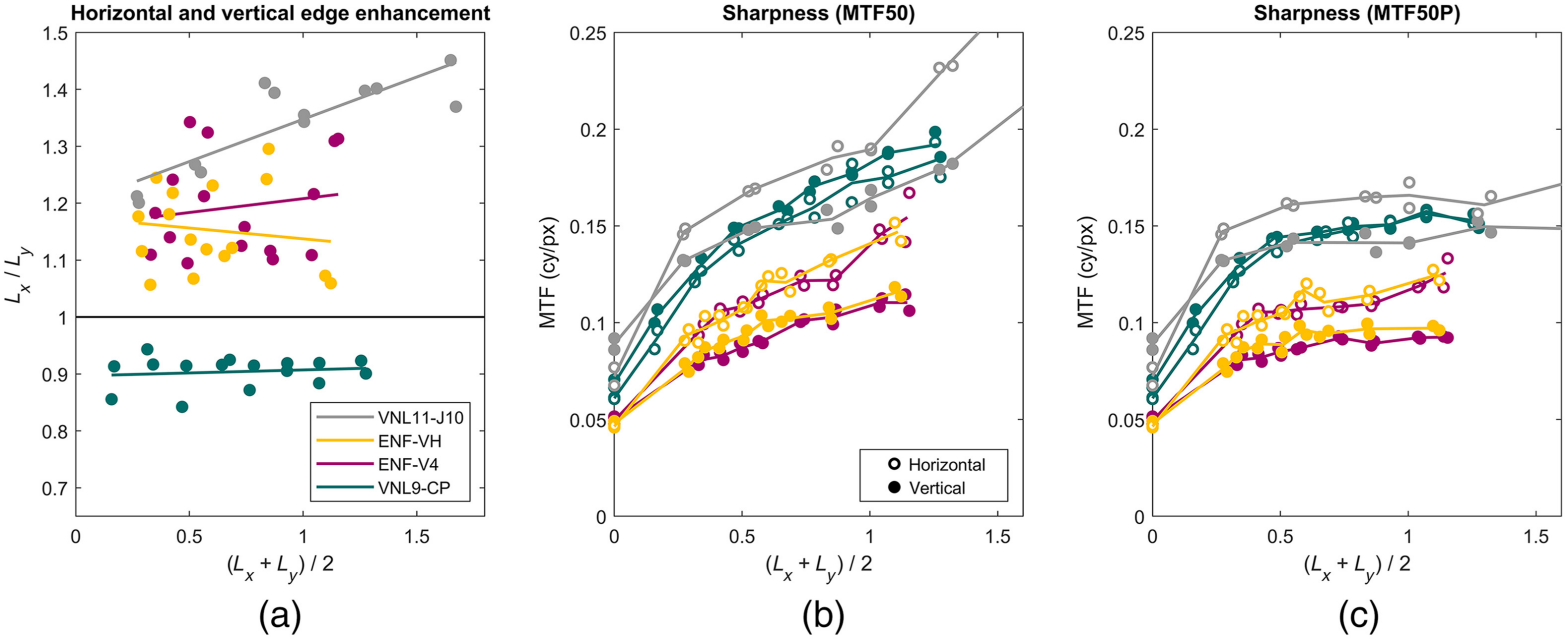
(a) Ratios of the horizontal level of edge enhancement $L_{x}$ and the vertical level $L_{y}$ are plotted versus the mean of $L_{x}$ and $L_{y}$. Ratios close to one indicate equal levels of horizontal and vertical edge enhancement. (b) Sharpness is measured as MTF50 expressed in units of cycles per pixel and keeps increasing when the level of edge enhancement is increased. The VNL9-CP has approximately equal horizontal and vertical MTF50 values. The MTF50-values of other endoscopes diverge when edge enhancment is applied. These differences in sharpness correspond to the difference between the horizontal and vertical level of edge enhancement. The Olympus MTF50-values of the ENF-V4 and ENF-VH overlap because they are processed at the same pixel resolution. (c) MTF50P starts exactly the same as MTF50, but MTF50P flattens off while MTF50 keeps increasing. This is useful for comparing the sharpness when the level of edge enhancement is unknown. It is worth noting that MTF50 should be converted to cycles per unit distance in order to be compared to other endoscopes.

The sharpness was measured by MTF50 [Fig. 11(b)] and MTF50P [Fig. 11(c)]. Figure 11 shows sharpness expressed in units of cycles per pixel. Horizontal and vertical sharpness are equal when no edge enhancement is applied, except for the VNL11-J10. Sharpness increases steeply as edge enhancement is applied and the horizontal and vertical sharpness start to diverge when edge enhancement is applied, except for the VNL9-CP. The difference in sharpness corresponds to the ratios of horizontal and vertical level of edge enhancement [Fig. 11(a)]. Olympus ENF-V4 and ENF-VH overlap, suggesting that the images are processed equally on the pixel level.

**Fig. 12:**
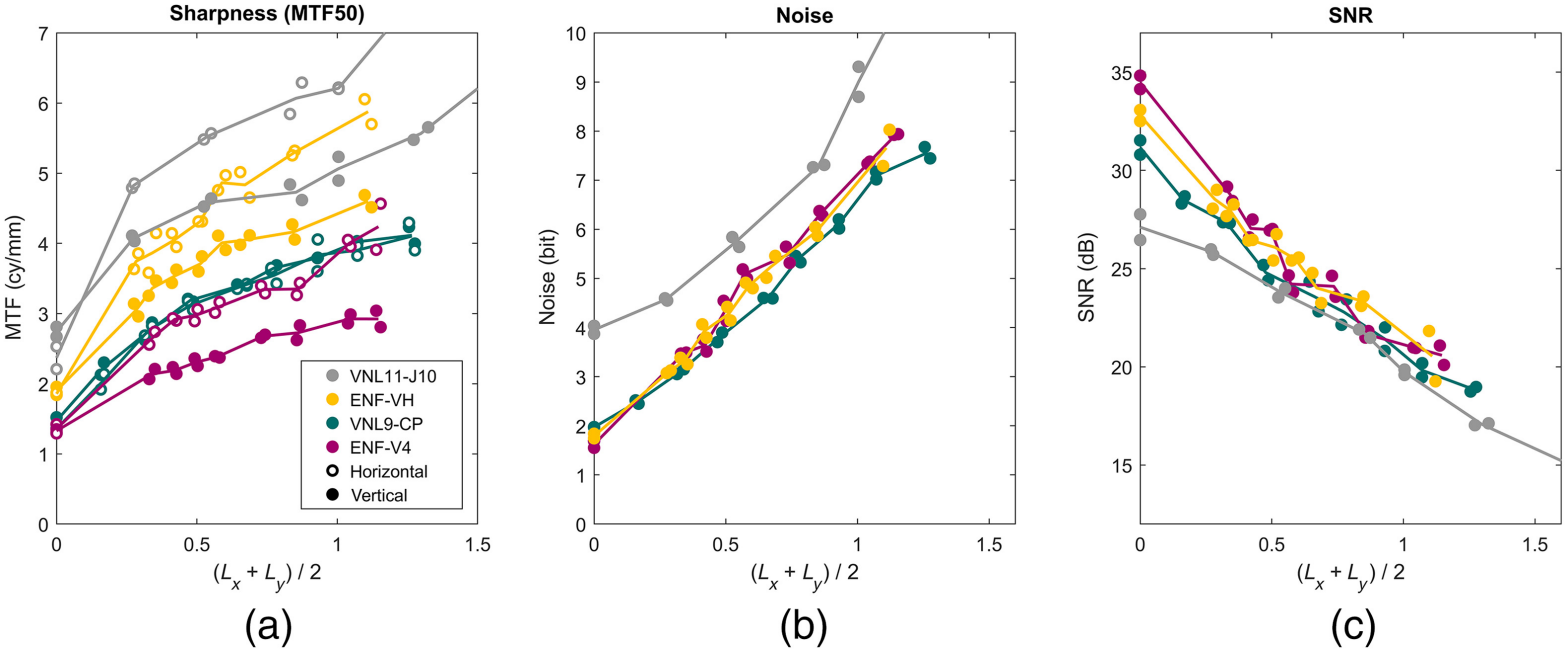
(a) Sharpness is measured as MTF50 expressed in units of cycles per mm so sharpness can be compared between endoscopes. The VNL9-CP and ENF-V4 have a very similar sharpness when the edge enhancement is switched off. As the level of edge enhancement increases, the vertical sharpness diverges from the horizontal ENF-V4 curve and VNL9-CP curves. The ENF-VH has a better sharpness compared to the ENF-V4 and VNL9-CP, but is exceeded by the VNL11-J10. The VNL11-J10 values are consistently larger than the other endoscopes, in particular the MTF values in cycle per mm without edge enhancement lie above the other endoscopes, indicating a smaller effective pixel size of the video chip. (b) Noise increases with higher levels of edge enhancement. The VNL11-J10 has considerable higher noise values. (c) Signal-to-noise measured in decibels. All endoscopes have similar SNR-values, with VNL11-J10 the lowest.

Figure 12(a) shows sharpness expressed in units of cycles per millimeter, which is suitable for comparing the level of detail that can be captured by the endoscopic systems. The VNL9-CP and ENF-V4 have a very similar sharpness when the edge enhancement is switched off. As the level of edge enhancement increases, the vertical sharpness of the ENF-V4 diverges from the horizontal ENF-V4 curve and VNL9-CP curves, indicating that the ENF-V4 vertical edge enhancement is softer. The ENF-VH has a better sharpness compared to the ENF-V4 and VNL9-CP but is exceeded by the VNL11-J10. The VNL11-J10 values are consistently larger than the other endoscopes, in particular the MTF values in cycle per mm without edge enhancement lie above the other endoscopes, indicating a smaller effective pixel size of the video chip. It can also be seen that the horizontal MTF50 of the VNL11-J10 starts below MTF50 vertical at zero edge enhancement, but MTF50 horizontal exceeds MTF50 vertical when edge enhancement is applied.

MTF50P [Fig. 11(c)] is introduced to counter the effect of edge enhancement when comparing sharpness of imaging systems. Although MTF50P starts off the same as MTF50, MTF50P flattens off for higher values of edge enhancement when the peak of the MTF curve grows larger than the contrast value at zero frequency while MTF50 keeps increasing. The MTF50P value could be useful for comparing the sharpness of endoscopic systems when the levels of edge enhancement are relatively large, but the exact levels are unknown. For example, if the ENF-V4 with a large level of edge enhancement is compared to the VNL9-CP with a medium level of edge enhancement, the measured sharpness of the ENF-V4 can exceed the sharpness of the VNL9-CP.

### 3.3 Noise

The noise values were measured at the gray patches 3–7, 9, 10, and 12 (see Fig. 9 for numbering). The patches 1 and 2 were excluded, due to saturated luminance values and the patches 8 and 11 were excluded as the endoscopes could resolve texture on those gray patches, and noise should be measured from a constant reflection surface. Figure 12(b) shows the measured noise values along the y axis. The noise increases linearly with the level of edge enhancement. The Pentax VNL9-CP has slightly lower levels of noise compared to the Olympus ENF-V4 and ENF-VH, and the VNL11-J10 has considerable higher noise levels.

**Fig. 13:**
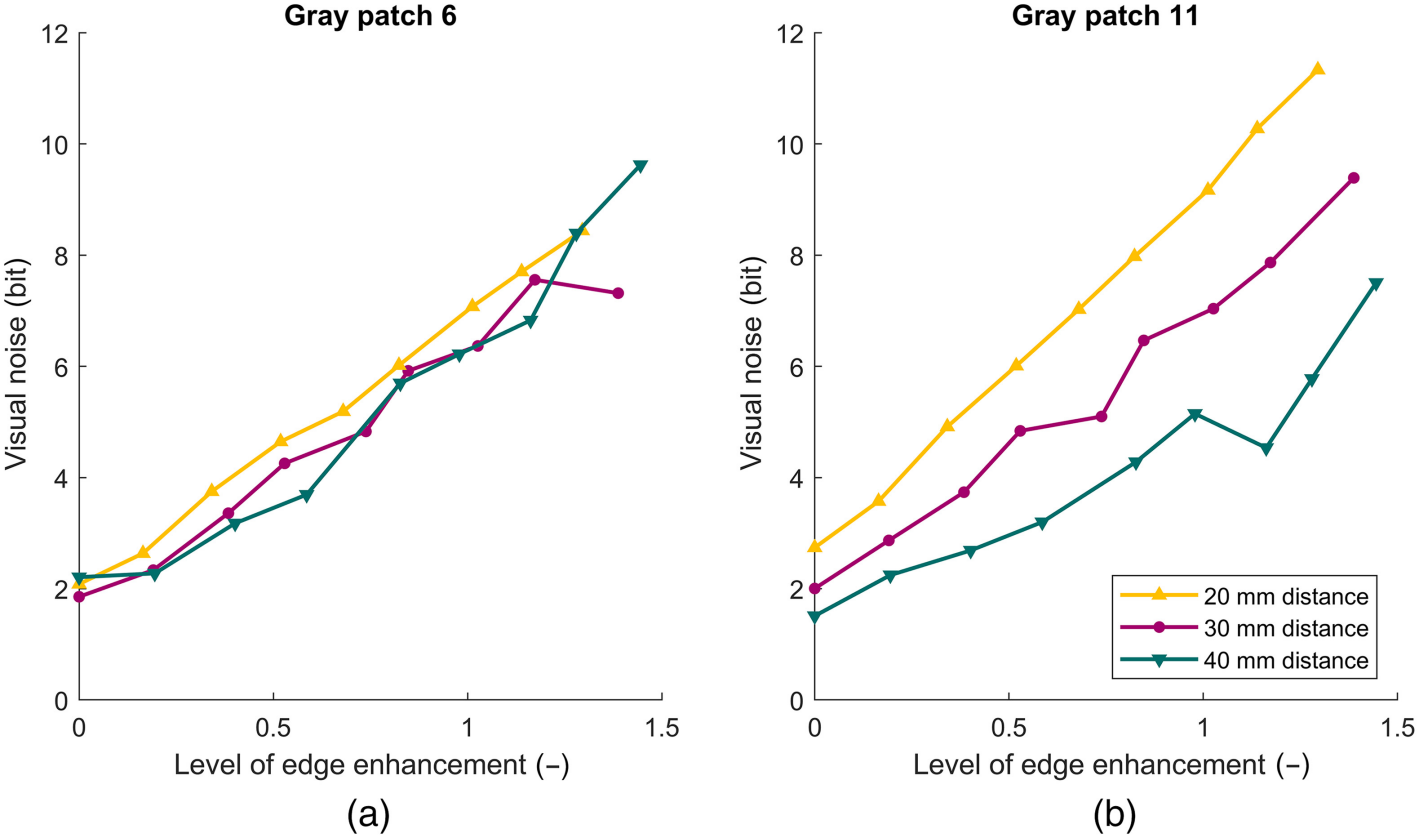
(a) Noise versus the level of edge enhancement measured at 20-, 30-, and 40-mm distance of the endoscope tip to the test chart. The curves for patch 6 overlap, indicating that the image is flat and that the endoscope is unable to resolve any texture and the surface can be used for noise measurements. (b) The noise measured at patch 11. The noise increases when the endoscope tip is positioned closer (20 mm) to the test target and decreases as the tip is moved further away (40 mm). This indicates that the endoscope can resolve texture on the surface, which adds up to the present noise, resulting in overestimation of the image noise.

Two additional series of images were acquired with the VNL9-CP to illustrate the influence of the endoscope tip to target distance and show how texture on the gray patches can affect noise measurements. Figure 13(a) shows the noise on patch 6 measured at a distance of 20, 30, and 40 mm between the tip of the endoscope and target. All curves overlap, indicating that the measured noise on patch 6 is independent of the distance. Figure 13(b) right shows the measured noise on patch 11. In contrast to patch 6, the noise increases when the endoscope is positioned closer (20 mm) to the test target and decreases as the tip is moved further away (40 mm). If an endoscope is able to resolve texture on a patch surface, this patch should be excluded for noise measurements. Comparing the measured noise between two distances can help in determining if texture is influencing the noise measurements (Fig. 13).

### 3.4 Signal-to-Noise Ratio

SNRs are measured in decibels [Fig. 12(c)]. The SNR-values decrease consistently as the level of edge enhancement is increased due to higher noise values. The VNL11-J10 starts with lower SNR-values, but as the level of edge enhancement increases, the SNR-values of all endoscopes become similar.

## 4 Discussion

In this study, we measured the level of edge enhancement using a method that can be applied to endoscopic systems with undisclosed methods of edge enhancement and we measured the effect of edge enhancement on sharpness and noise. The measured level of edge enhancement correlates at large with the ordinal levels set at the user interface of the video processor. The horizontally and vertically found edge enhancement can differ. If the edge enhancement is switched off, we find no difference in sharpness, indicating that our method is orientation insensitive and that the pixels of the different endoscopes are square. We speculate that the implementation of the edge enhancement algorithm on the video processors is rotational assymmetric in favor for faster execution times. That is, small kernel sizes for derivatives filters perform poorly but are a computationally faster approximation, as images need to be displayed without perceived delay.

Sharpness, measured as MTF50, increases with applied edge enhancement. Our results on sharpness are consistent with Koren who reported MTF50 and MTF50P values for images with a known edge enhancement algorithm and parameters.^18^ The consistency with the results obtained with a known method and parameters for edge enhancement confirms that the presented method for unknown method and parameters works. The prerequisite for the presented method to work is that there is an option to switch the edge enhancement between different levels.

For a fair visual and objective (MTF50) comparison of sharpness, edge enhancement should be taken into account. In our previous study,^7^ 30 ENT-professionals compared *in vivo* images of one larynx captured using the (edge enhancement) settings as recommended by the vendors. About 28 Observers preferred the VNL9-CP (recommended setting 50% edge enhancement) over the ENF-V4 (enhancement setting A1) and the measured MTF50-values were 3.58 versus 2.15 lp/mm, respectively. From the result presented above, however, we know that both systems have an identical horizontal sharpness curve as function of edge enhancement (though vertical sharpness of the ENF-V4 is 25% poorer than the VNL9-CP, compare Fig. 12). The results of the previous study could have been different, if the absolute amount of sharpening would have been used.

As expected, the induced noise increases with the level of edge enhancement as the algorithms cannot discern information in the image from noise. The relative importance of induced noise compared to sharpness in clinical practice is still to be determined. For the moment, we have two qualities that cannot directly be combined into one number for a device.

We ask why edge enhancement is needed or applied at all. If the image is properly sampled according to Nyquist, edges are already perceived as steep with a transition of about σ=1 pixel of Gaussian blurring, e.g., for the VNL9-CP we took the endoscope apart and inspected the camera chip on the tip under a digital microscope and found that the chip has 400×400  pixels (with Bayer filter layer on the chip for RGB acquisition). Therefore, the displayed image of 800×800  pixels needs first to be demosaiced from the Bayer pattern and then enlarged by a factor of 2. We speculate that this is similar for other types of endoscopes and thus images without edge enhancement are blurred by the interpolation of the physical to the displayed number of pixels [compare Figs. 9(a) and 9(b)]. This initial blurring due to upsampling can be mitigated partially by the edge enhancement.

Our next step is designing a study that involves comparing *in vivo* images with different levels of edge enhancement to explore its relation to the perceived image quality by ENT-professionals. There probably is an optimum for the level of edge enhancement with respect to the perception by ENT-professionals. Images with low levels of edge enhancement will be perceived as vague, whereas excessive levels of edge enhancement yield sharper images but contain objectionable artifacts and too much noise. A study on the optimal level of edge enhancement could substantiate a default setting for ENT-endoscopic systems that is independent of the vendor and can also be used for future generation endoscopes.

## 5 Conclusions

The presented method overcomes the issue of vendors expressing the level of edge enhancement each differently in arbitrary units. This allows us to compare the effect of edge enhancement across different vendors, and we can start exploring the relationship between those effects and the subjectively perceived image quality by ENT-professionals and find and recommend substantiated optimal settings.

## Code, Data, and Materials Availability

Matlab code for analyzing the Rez checker target can be downloaded from: https://github.com/GGeleijnse/OnTheEdge.
