# Influence of edge enhancement applied in endoscopic systems on sharpness and noise (Erratum)

**DOI:** 10.1117/1.JBO.28.7.079801

**Published:** 2023-07-21

**Authors:** Geert Geleijnse, Bernd Rieger

**Affiliations:** a Erasmus University Medical Center, Department of Ear, Nose, & Throat, Rotterdam, The Netherlands; b Delft University of Technology, Department of Imaging Physics, Faculty of Applied Sciences, Delft, The Netherlands

## Abstract

The erratum corrects a typo in Eq. (4) of the published article.

This article [*J. Biomed. Opt.* **27**(10), 106001 (2022) doi: 10.1117/1.JBO.27.10.106001 was originally published on 6 October 2022 with an error in Equation 4.

Original

$$\sigma_{V}(Y,R,G)=\sqrt{\sigma {\left(Y\right)}^{2}+0.278\cdot \sigma {\left(Y-R\right)}^{2}+0.088\cdot \sigma {\left(Y-G\right)}^{2}}$$

Corrected

$$\sigma_{V}(Y,R,B)=\sqrt{\sigma {\left(Y\right)}^{2}+0.279\cdot \sigma {\left(Y-R\right)}^{2}+0.088\cdot \sigma {\left(Y-B\right)}^{2}}$$

The authors checked their software and found that they had used the correct equation there, so the reported results were unaffected by the typo. The article was corrected and republished on 8 July 2023.

